# Functional characterization of a liverworts bHLH transcription factor involved in the regulation of bisbibenzyls and flavonoids biosynthesis

**DOI:** 10.1186/s12870-019-2109-z

**Published:** 2019-11-14

**Authors:** Yu Zhao, Yu-Ying Zhang, Hui Liu, Xiao-Shuang Zhang, Rong Ni, Piao-Yi Wang, Shuai Gao, Hong-Xiang Lou, Ai-Xia Cheng

**Affiliations:** Key Laboratory of Chemical Biology of Natural Products, Ministry of Education, School of Pharmaceutical Sciences, Shandong University, Jinan, 250012 China

**Keywords:** bHLH transcription factor, Liverworts, Bisbibenzyls, Flavonoids, *P. appendiculatum*

## Abstract

**Background:**

The basic helix-loop-helix (bHLH) transcription factors (TFs), as one of the largest families of TFs, play important roles in the regulation of many secondary metabolites including flavonoids. Their involvement in flavonoids synthesis is well established in vascular plants, but not as yet in the bryophytes. In liverworts, both bisbibenzyls and flavonoids are derived through the phenylpropanoids pathway and share several upstream enzymes.

**Results:**

In this study, we cloned and characterized the function of PabHLH1, a bHLH family protein encoded by the liverworts species *Plagiochasma appendiculatum*. PabHLH1 is phylogenetically related to the IIIf subfamily bHLHs involved in flavonoids biosynthesis. A transient expression experiment showed that PabHLH1 is deposited in the nucleus and cytoplasm, while the yeast one hybrid assay showed that it has transactivational activity. When PabHLH1 was overexpressed in *P. appendiculatum* thallus, a positive correlation was established between the content of bibenzyls and flavonoids and the transcriptional abundance of corresponding genes involved in the biosynthesis pathway of these compounds. The heterologous expression of PabHLH1 in *Arabidopsis thaliana* resulted in the activation of flavonoids and anthocyanins synthesis, involving the up-regulation of structural genes acting both early and late in the flavonoids synthesis pathway. The transcription level of PabHLH1 in *P. appendiculatum* thallus responded positively to stress induced by either exposure to UV radiation or treatment with salicylic acid.

**Conclusion:**

PabHLH1 was involved in the regulation of the biosynthesis of flavonoids as well as bibenzyls in liverworts and stimulated the accumulation of the flavonols and anthocyanins in Arabidopsis.

## Background

**Fig. 1:**
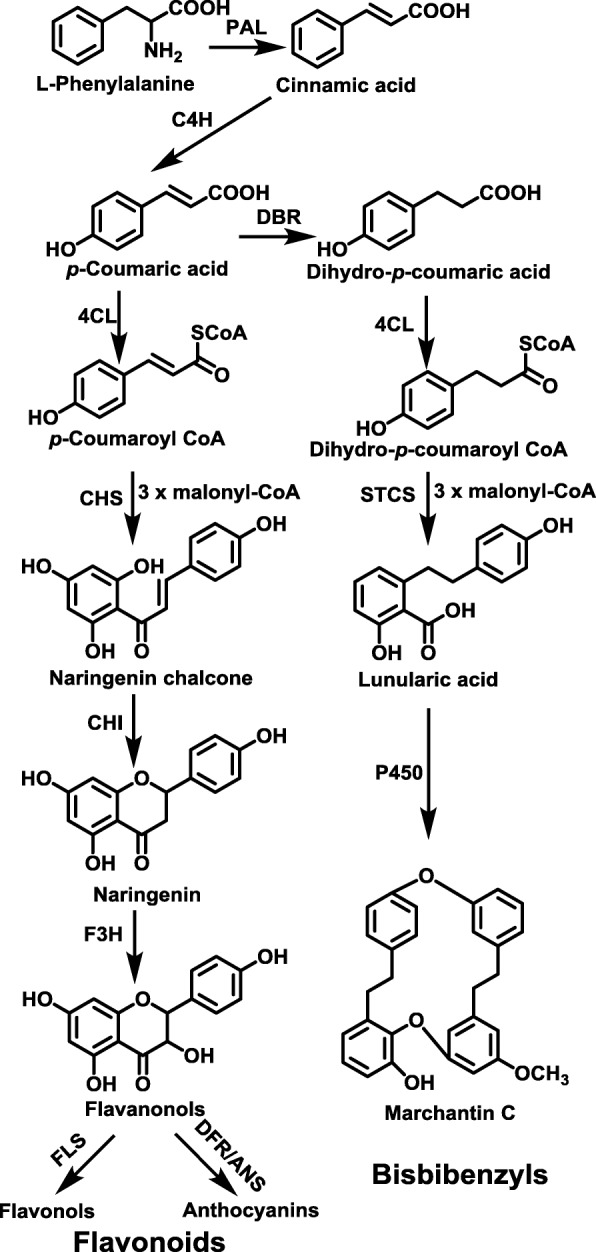
The Schema of plant flavonoids biosynthesis. *PAL* Phenylalanine ammonia lyase, *C4H* Cinnamic acid 4-hydroxylation, 4CL: 4-coumarate: coenzyme A ligase, CHS: chalcone synthase, CHI: chalcone isomerase, F3H: flavanone 3β-hydroxylase, FLS: flavonol synthase, DFR: dihydroflavonol reductase, ANS: anthocyanidin synthase. DBR: double bond reductase, STCS: stilbenecarboxylate synthase

Liverworts, which belong to a sub-group of the non-vascular bryophytes, produce a variety of secondary metabolites, including bisbibenzyls [1, 2], flavonoids [3, 4] and terpenoids [5]. A number of simple flavonoids, such as flavone *C*- or *O*-glucosides, flavones, trihydroxyflavanones, and isoflavonoids (e.g., 5, 3′, 4′-trihydroxyisoflavone-7-*O* glucoside) are found in the liverworts [3, 4]. The bibenzyls and cyclic bis (bibenzyls) are the predominant phenolic compounds produced by liverworts [6]. Prominent examples are the bibenzyl lunularic acid, its decarboxylation product lunularin and various coupled cyclic bis (bibenzyl) compounds [6]. These substances contain two bibenzyl moieties which are condensed by ether bridges and/or C-C bonds [6] and exhibit pronounced anti-fungal and anti-tumor activity [7, 8]. As has been shown repeatedly in a number of higher plant species, flavonoids are synthesized via the phenylpropanoid and polyketide pathway. The bisbibenzyls are also derived through the phenylpropanoid and polyketide pathway, beginning with the L-phenylalanine and via cinnamic acid, *p*-coumaric acid, dihydro-*p*-coumaric acid to form dihydro-*p*-coumaryl CoA [9]. The condensation of dihydro-*p*-coumaryl CoA with three of malonyl-CoA forms lunularic acid; when catalyzed by cytochrome P450 monooxygenase, two lunularic acid molecules condense to yield bis-bibenzyl [9]. The biosynthesis of the bis-bibenzyls and the flavonoids share several upstream steps in liverworts [10, 11] (Fig. 1).

In plants, the structural genes responsible for flavonoids biosynthesis are regulated largely at the transcriptional level, controlled by two classes of transcription factors (TF) (MYB and bHLH) along with the WD40 proteins. The bHLH TFs are essential for the activity of the R2R3 MYB partner, and also provide enhanced activity on promoters containing a cis-regulatory element conserved in several flavonoids and anthocyanins biosynthetic genes [12]. The bHLH family has been divided into 26 sub-groups [13], which regulate a wide range of cellular processes, including the fate of epidermal cells, the hormonal response, metal homeostasis, photomorphogenesis and floral organ development [14]. The flavonoids biosynthesis related bHLHs have been assigned to a subgroup denoted IIIf. One of the first identified members of the subgroup is the maize *R* gene, which encodes a regulator of anthocyanins accumulation in the grain [15]. The delila (del) gene encoding bHLH factor regulates the pattern of red anthocyanin pigmentation in *Antirrhinum majus* plants [16]. The *Arabidopsis thaliana* bHLH proteins AtTT8, AtGL3 and AtEGL3 are all involved in the synthesis of various flavonoids [17–19]. ANTHOCYANIN1 (AN1) of petunia is a bHLH transcription factor that is required for the synthesis of anthocyanin pigments [20]. In rice, the products of *Rc* and *Rd* are bHLH transcription factors which control proanthocyanidins synthesis in the grain pericarp, while the tobacco genes *NtAn1a* and *NtAn1b* encode enhancers of anthocyanin accumulations in the flower [21]. In *Dahlia variabilis,* DvIVS (a member of the An1 subgroup of bHLH transcription factors) is involved in the regulation of anthocyanins synthesis in the ray florets [22]. In morning glory, IpIVS regulates both proanthocyanidins accumulation in seeds and anthocyanins biosynthesis in flowers [23]. The two grapevine bHLH proteins VvMYC1 and VvMYCA1 are required for the production of, respectively, anthocyanins and proanthocyanidins [24, 25].

The regulation of the flavonoids synthesis by bHLH TFs has been extensively studied in vascular plants, but as yet, little is known concerning their participation in the regulation of bisbibenzyls and flavonoids synthesis in the liverworts. Liverworts represent a division of bryophyte species and are among the earliest diverging lineages of land plants dated to the early Ordovician *period* (about 488 million to 444 million years ago). Characterization of the bHLH genes involved in the regulation of flavonoids biosynthesis from liverworts will shed light on the elucidation of the origin and evolution of bHLH transcript factors. In our previous investigation, PabHLH has been isolated and functional characterized as a positive regulator of bisbibenzyls biosynthesis from liverworts *P. appendiculatum* [26]. Here, we characterized another bHLH (PabHLH1) gene from *P. appendiculatum* by over-expressing in *P. appendiculatum* and heterologously expressing in *A. thaliana*. PabHLH1 was positively regulated the bisbibenzyls and flavonoids biosynthesis in *P. appendiculatum* and stimulated the accumulation of the flavonols and anthocyanins in Arabidopsis.

## Methods

### Plant materials

*P. appendiculatum* thallus (A little bit samples were originally collected from Sichuan province, China and then cultured and propagated in our green house, and authenticated by Professor Xuesen Wen. A voucher specimen with No. 20091010–01 has been deposited in the School of Pharmaceutical Sciences, Shandong University. No specific permits were required for collecting this sample for research purpose.) was raised in green house delivering a constant temperature of 25 °C and a 12 h photoperiod in Shandong University. Two-month-old thallus were harvested and snap-frozen in liquid nitrogen and stored at − 80 °C. Axenic thallus and callus were raised, respectively, on half strength Murashige and Skoog (1962) (MS) medium [27] supplemented with 0.5 mg L^− 1^ 6-benzyladenine. The cultures were exposed to a 12 h photoperiod and a day/night temperature regime of 22 °C/20 °C. *A. thaliana* ecotype Col-0 (purchased from Arabidopsis Biological Resource Center) plants were raised in a growth chamber under a 16 h photoperiod at a constant temperature of 22 °C. The *Nicotiana benthamiana* (Donated by Professor Fengning Xiang from School of life sciences, Shandong University) plants required for transient expression experiments were soil-grown for 5–6 weeks under a 12 h photoperiod and a day/night temperature regime of 24 °C/22 °C.

### Nucleic acid extraction and gene isolation

Total RNA was extracted from *P. appendiculatum* thallus or *A. thaliana* seedlings using, respectively, a CTAB-based method [28] and the RNAiso Plus reagent (TaKaRa, Kusatsu, Shiga, Japan). cDNA was synthesized from preparations of total RNA using PrimeScript™ RT Master Mix (Takara, Otsu, Japan), according to the manufacturer’s protocol. A *PabHLH1* fragment lacking the full coding region was identified from *P. appendiculatum* transcriptome sequencing database (SRP073827). The 3′-RACE method was then applied to recover the missing 3′ sequence: the template for this reaction was 3′-Ready cDNA and the primer pair was PabHLH1-NGP (sequences given in Additional file 1: Table S1), along with the UPM primer provided in the SMART RACE cDNA Amplification kit (Clontech, USA). Once the complete open reading frame (ORF) sequence of *PabHLH1* had been acquired, it was amplified from *P. appendiculatum* thallus cDNA using the primer pair PabHLH1-F/R (sequences given in Additional file 1: Table S1); the amplicon was then inserted into pMD19-T and sequenced for validation purposes.

### Sequence analysis

The predicted PabHLH1 polypeptide sequence was aligned with those of its plant homologs *A. thaliana* TT8 (CAC14865), *Ipomoea purpurea* IpIVS (BAD18982) and *Vitis vinifera* MYC1 (EU447172) using DNAMAN v5.2.2 software (Lynnon Biosoft, Quebec, Canada). A phylogenetic analysis was conducted based on the maximum likelihood method implemented in MEGA v4.0 software [29], based on 1000 bootstrap replicates.

### Stress treatment of *P. appendiculatum* thallus

One hundred two month old *P. appendiculatum* thallus were irradiated with a UV-B 311 nm narrow band lamp (Philips, PL-S 9 W/01/2P, Poland) (60 mJ/cm^2^) or 1 mM salicylic acid (SA) for 10 min, and then harvested after 0, 6, 12, 24, 36, 48 and 60 h. The material was snap-frozen in liquid nitrogen and stored at − 80 °C until processed for qRT-PCR assays as described above.

### Quantitative real time PCR (qRT-PCR) analysis

The qRT-PCR approach was used to characterize the transcriptional behavior of *PabHLH1* in three different tissues. The expression of *PabHLH1* along with that of a number of phenylpropanoid pathway genes, flavonoid structural genes and the bibenzyl synthesis genes were analyzed using qRT-PCR in both wild type and transgenic *P. appendiculatum* and *A. thaliana*. The required cDNA template was derived from RNA extracts as described above, and the qRT-PCRs were performed using an Eppendorf Mastercycler ep realplex RealTime PCR System (Eppendorf, Germany). The relevant primers are listed in Additional file 2: Table S2. Each 10 μL reaction comprised 2 μL SYBR, 1 μL of the template cDNA, 0.5 μL of each primer pair (10 μM) and 6.5 μL RNase-free dH_2_O. The reference gene for the *P. appendiculatum* samples encodes an elongation factor, as amplified by the primer pair *P. appendiculatum* elongation F/R [30]. All samples were evaluated in three independent experiments.

### Subcellular localization and transcriptional activity assay

The full length ORF (lacking the stop codon) was PCR-amplified using primers including an attB recombination site (sequences given in Additional file 1: Table S1), and the amplicon was subjected to a BP clonase reaction in order to insert it into the vector pDONR207 (Invitrogen, Carlsbad, CA, USA). Recombined plasmids were processed in a Gateway LR clonase reaction (Invitrogen, Carlsbad, CA, USA) with pGWB5 vector to create a PabHLH1-GFP fusion driven by the CaMV 35S promoter [31]. The construct was transferred into *Agrobacterium tumefaciens* strain EHA105 using the freeze/thaw method and from thence into *N. benthamiana* epidermal leaf cells or onion epidermal cells [26] for its transient expression. The onion epidermal cells were incubated with 0.1 mg ml^− 1^ DAPI for 10 min for staining the nucleus. The GFP and DAPI fluorescence was monitored via confocal laser scanning microscopy (LSM 700, Carl Zeiss, Inc., New York, USA) or fluorescence microscope (Olympus IX71; Olympus Co., Tokyo, Japan). Four different segments of *PabHLH1* were generated by *Nde* I-*Bam*H I or *Nco* I-*Bam*H I restriction to produce gene fragments corresponding to residues 1–261, 262–460, 461–702 and 1–460 of the full polypeptide sequence; these were ligated separately to the pGBKT7 plasmid (Clontech, USA). The full-length sequence of PabHLH1 lacks the available restriction endonuclease sites, so a single nucleotide substitution (a957c) was created using the Stratagene QuickChange site-directed mutagenesis method. The necessary mutant primers designed by primer X software (www.bioinformatics.org/primerx) (Table S1). The resulting PCR product was gel-purified and digested with Dpn I (Thermo Scientific, USA) at 37 °C for 4 h, and an aliquot was transformed into *E.coli* DH5α. After sequenced, mutated gene (PabHLH1-a957c) was inserted into the pGBKT7 (Clontech, USA) vector after digested with Nde I and Nco I (the primers used in this study are listed in Table S1). Each construct was then transformed into yeast strain AH109 using the PEG/LiAC method [32]. Yeast transformants were cultured on a synthetic drop-out medium lacking tryptophan (SD-Trp). After their PCR-based validation, transformed colonies were tested for the ability to growth on triple selection SD medium lacking tryptophan, histidine and adenine (SD-Trp-His-Ade).

### Plant transformation

The *PabHLH1* coding sequence harbored within pDONR207 was placed in pGWB5 vector under the control of the CaMV 35S promoter by the Gateway cloning technique [32], then introduced into *A. tumefaciens* EHA105. A 1 mL aliquot of an overnight *A. tumefaciens* culture was inoculated into 100 mL yeast extract peptone medium containing 50 mg/L kanamycin, and held at 30 °C with shaking until the OD_600_ had reached 1.5–2.0. The cells were harvested by centrifugation (4000 *g*, 5 min), then re-suspended in 50 mL liquid half strength MS medium (pH 5.2) containing 100 μM acetosyringone (Sigma-Aldrich, St. Louis, MO, USA). About fifty *P. appendiculatum* thallus were chopped into small pieces and incorporated into the *A. tumefaciens* suspension. The mixture was held for 1 h with shaking, then cultured for 72 h in the dark at 22 °C on solid half strength MS medium (pH 5.6–5.8) containing 100 μM acetosyringone. The thallus pieces were rinsed three times in sterile deionized water, then transferred to a half strength MS medium containing 25 mg/L hygromycin and 200 mg/L cefotaxime. After 2–3 weeks, surviving thallus were sub-cultured on half strength MS medium containing 50 mg/L hygromycin and 50 mg/L cefotaxime, with the medium being refreshed at two weekly intervals. *A. thaliana* ecotype Col-0 was transformed with *A. tumefaciens* GV3101 harboring the transgene using the flower dip method [33]. Regenerated plants were raised at 22 °C under a 16 h photoperiod, and putative T_1_ transformants were PCR-validated using the primer pair PabHLH1-vector-F/−R (sequences given in Additional file 1: Table S1).

### Compositional analysis of *P. appendiculatum* thallus

Transgenic and non-transgenic *P. appendiculatum* thallus, grown on half strength MS medium for about 20 days, was freeze-dried. A 25 mg aliquot was suspended in 500 μL methanol and ultrasonicated for 1 h to extract the bibenzyls fraction (baicalein was added as the internal standard). After centrifugation, a 20 μL volume of supernatant was separated via a reverse-phase Luna 5u C18(2) 100A column (4.6 × 250 mm, Phenomenex, CA, USA) using Agilent 1260 series system (Agilent, CA, USA). The HPLC parameters were: solvent A: 0.1% (v/v) aqueous formic acid, solvent B: acetonitrile; the solvents were provided at a flow rate of 0.8 mL/min. The initial solvent mix was 60% A/40% B, followed by 45 min of 33% A/67% B, then 5 min of 10% A/90% B, and finally 5 min of 100% B. The detection wavelength was 280 nm. The major bibenzyls, lunularic acid, riccardin C and riccardin D, were identified on the basis of corresponding standard samples and quantified according to the standard curve of the corresponding compound. For flavonoids analysis, 10 mg freeze-dried thallus was suspended in 800 μl 80% methanol and ultrasonicated for 1 h (myricetin was added as the internal standard). Flavonoids content was determined as aglycones by preparing acid-hydrolyzed extracts. An aliquot of 400 μL of the supernatant was acid-hydrolyzed by adding 120 μL of 3NHCl, incubated at 90 °C for 1 h, and then mixed with 200 μL of methanol. An Agilent 1260 series HPLC system equipped with a Luna 5u C18(2) 100A column (4.6 × 250 mm, Phenomenex, CA, USA) was used for chromatographic analysis. The HPLC conditions were as follows: the mobile phase consisted of solvent A (acetonitrile) and solvent B (1% acetic acid in water, v/v). The gradient elution program was: 0–10 min, 30% solvent A; 10–30 min, 30–45% solvent A; 30–35 min, 45–100% solvent A; 35–45 min, 100–30% solvent A. The detection wavelength was set at 330 nm. The flavonoid was quantified as the relative content in wild type and transgenic *P. appendiculatum* thallus according to the peak area. All samples were evaluated in three independent experiments.

### Flavonoids and lignin determination in transgenic *A. thaliana*

Ten day old transgenic and wild type (WT) *A. thaliana* seedlings were freeze-dried and ground to fine powder. For the determination of flavonoids composition, a 15 mg aliquot of the powder was extracted by sonication (1 h) in 800 μL methanol:water (80:20, v/v) [34]. Following centrifugation (16,000 *g*, 4 °C, 15 min), the supernatant was subjected to reverse phase HPLC using an Luna 5u C18(2) 100A column (4.6 × 250 mm, Phenomenex, CA, USA). The mobile phase was 5–85% acetonitrile and 95–15% water (0.1% v/v formic acid) provided at a flow rate of 0.8 mL/min over 40 min. The detection wavelength was 364 nm. Peaks were identified as specific kaempferol derivatives using MS and UV spectral analysis [34]. Chrysin was used as the internal standard to normalize the peaks and calculate the relative contribution of the various flavonols. Seedlings were visually screened for anthocyanin accumulation after 5 days of culture on anthocyanin gene induction media half strength MS (pH 5.8) consisting of sucrose (3%, w/v) and agar (0.8%, w/v). For the total anthocyanins analysis of transgenic and wild type lines, 100 mg fresh plant material was suspended in 400 μL methanol with 1% HCl (v/v) and ultrasonicated for 1 h. After centrifugation (15,000 g, 10 min), the supernatant was transferred to a fresh tube and the total anthocyanin was determined by measuring the OD at A530 and A657 by using a spectrophotometer (Shimadzu, Kyoto, Japan). The quantity of anthocyanin was determined by calculating absorbance A = (A530–0.25 × A657). The concentration of anthocyanin pigment in the original sample was calculated using the following formula: anthocyanin pigment (mg/L) = (A × MW × DF × 1000)/(ε × 1), where MW is the molecular weight of cyandindin-3-glucoside (449.2), DF is the dilution factor, and ε is the molar absorptivity of cyanindin-3-glucoside (26,900) [35]. The pattern of lignin distribution present in five- to seven-leaf stage plants was obtained by staining stem sections following the previous procedure [36]. The lignin content of the stem tissue was quantified by spectrophotometric (280 nm) analysis of an acetyl bromide extract. The samples were ground into powder in liquid nitrogen and extracted, in turn, with 70% ethanol, chloroform/methanol (1:1 v/v) and acetone for cell wall preparation. According to the acetyl bromide method [37], 6 mg dried samples were digested with 1 mL 25% acetyl bromide in 70 °C for 30 min, and sequentially added 5 mL acetic acid after cooled in an ice bath for 10 min. Then 300 μL supernatants were mixed by 400 μL of 1.5 M NaOH and 300 μL of 0.5 M hydroxylamine hydrochloride, followed 1.5 mL acetic acid to dilute. The extinction coefficient for *A. thaliana* lignin was 23.35 g^− 1^ L cm^− 1^ at 280 nm. All samples were evaluated in three independent experiments.

## Results

### The *PabHLH1* sequence

**Fig. 2:**
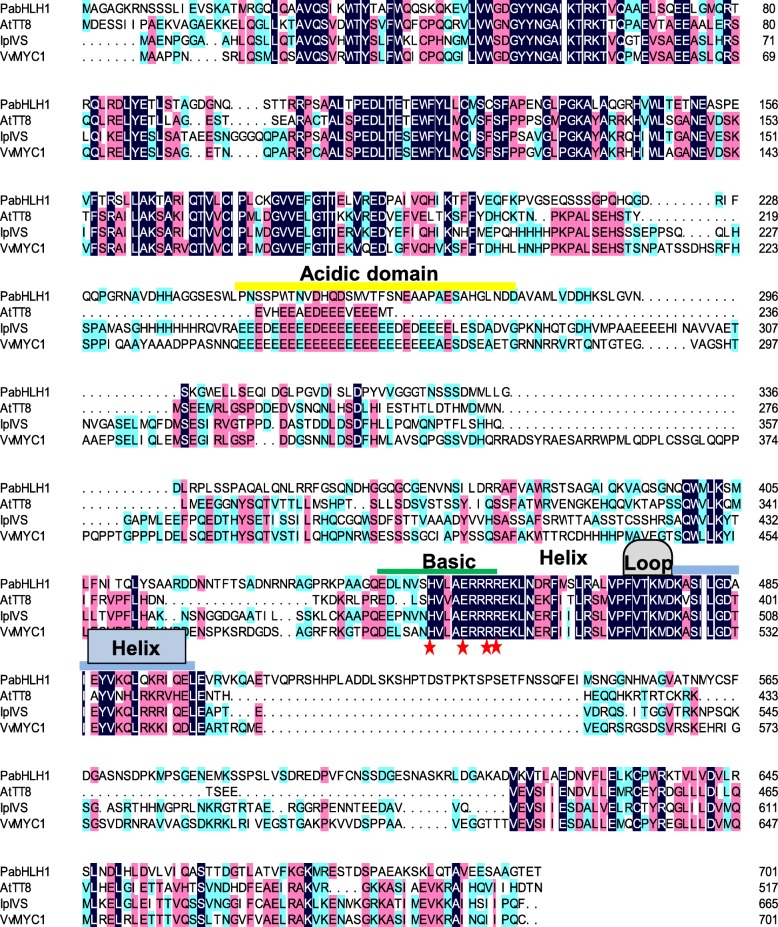
Sequence alignment of PabHLH1 and its closest homologs *Arabidopsis thaliana* TT8 (CAC14865), *Ipomoea purpurea* Ivory Seed (BAD18982) and *Vitis vinifera* MYC1 (EU447172). Residues composing the HER motif are shown with red stars. The bHLH domains and the acidic domain are labeled

**Fig. 3:**
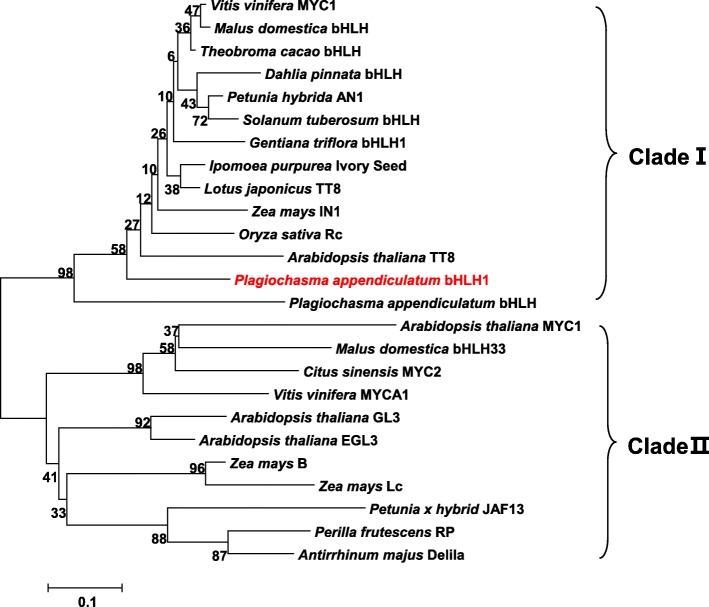
Phylogenetic tree of PabHLH1 and other plant bHLH TFs domains. The tree was constructed by the MEGA v4.0 program using neighbor-joining method. TFs sequences used are *Vitis vinifera* MYC1 (EU447172), *Malus domestica* bHLH (ADL36597), *Theobroma cacao* bHLH (XP_007050639.1), *Dahlia pinnata* bHLH (BAM84239), *Petunia hybrida* AN1 (AAG25927), *Solanum tuberosum* bHLH (AGC31677.1), *Gentiana triflora* bHLH1 (BAH03387), *Ipomoea purpurea* Ivory Seed (BAD18982), *Lotus japonicus* TT8 (BAH28881), *Zea mays* IN1 (AAB03841), *Oryza sativa* Rc (BAF42668), *Arabidopsis thaliana* TT8 (CAC14865), *Plagiochasma appendiculatum* bHLH (MF983804); *Plagiochasma appendiculatum* bHLH1 (AXO67713); *Arabidopsis thaliana* MYC1 (NP_001329692.1), *Malus domestica* bHLH33 (ABB84474), *Citus sinensis* MYC2 (ABR68793), *Vitis vinifera* MYCA1 (EF193002); *Arabidopsis thaliana* GL3 (NP_680372), *Arabidopsis thaliana* EGL3 (NP_176552.1), *Zea mays* B (CAA40544), *Zea mays* Lc (NP_001105339), *Petunia hybrid* JAF13 (AAC39455), *Perilla frutescens* RP (BAA75513), *Antirrhinum majus* Delila (AAA32663)

The full length ORF of *PabHLH1* was recovered from the partial transcript via 3′-RACE. Its length was 2109 bp and its predicted product was a 702 residue protein of molecular mass 76.86 KDa and pI 6.03. The predicted polypeptide’s sequence shared 27.9, 28.7 and 30.9% identity with its closest homologs AtTT8, IpIVS and VvMYC1, respectively (Fig. 2); the latter three proteins are known to regulate anthocyanin synthesis. Despite the relatively low overall level of sequence identity, the PabHLH1 sequence has retained the bHLH domain DNA binding HER motif (including His/Lys9, Glu13 and Arg17) [38, 39]. In the N terminal region, a high level of homology was noted within the MYB interaction region (MIR). In addition, the protein has retained the K165 and A167 residues shown to be essential for transcriptional activation [40]. A phylogenetic analysis involving the sub-group IIIf of plant bHLHs divided the proteins into two clades (Fig. 3): clade I included AtTT8, maize IN1, grapevine MYC1 and rice Rc, all of which are involved in the regulation of flavonoids biosynthesis, while clade II included transcription factors Petunia JAF13, Arabidopsis GL3, Snapdragon Delila (Del) and Maize Lc. The PabHLH1 sequence, along with the previous reported PabHLH, clustered within clade I and located at the peripheral position in the clade, which suggested that PabHLH and PabHLH1 transcript factor in the basal land plant liverwort likely represent an ancestral outgroup of the homologs of vascular plants.

### Expression of PabHLH1 in different tissues and its response to stress

**Fig. 4:**
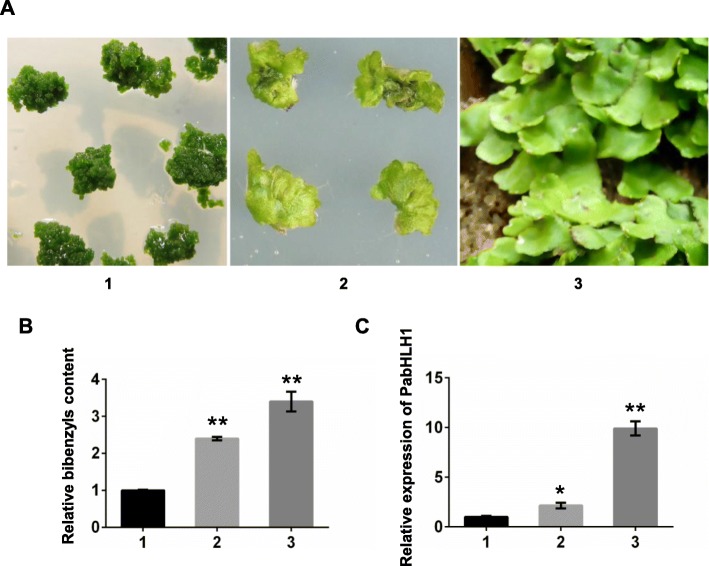
The relative bisbibenzyls content and the expression level of PabHLH1 in *P. appendiculatum*. **a** Three different tissues of *P. appendiculatum*, 1, 2: axenic callus and thallus cultured on MS medium; 3: thallus growing in greenhouse. **b** Relative bisbibenzyls content in three tissues referred to in (**a**). **c** PabHLH1 transcript abundance by qRT-PCR in the three tissue types referred to in (**a**). Data represent mean ± S.D. with three biological repeats. *p < 0.05, ** *p* < 0.01 according to Student’s t-test

To investigate the association between the expression profile of PabHLH1 and the accumulation of target metabolites, the bisbibenzyl contents and the expression pattern of *PabHLH1* in three different tissues, axenic callus, thallus and wild thallus growing in soil in greenhouse (Fig. 4a), were quantified. The bisbibenzyl content of thallus growing in greenhouse was highest and that of axenic thallus was higher than in the callus (Fig. 4b). At the same time, the expression pattern of *PabHLH1* was demonstrated showing consistently expressed tendency in the three different liverwort tissues with the bisbibenzyl contents (Fig. 4c).

**Fig. 5:**
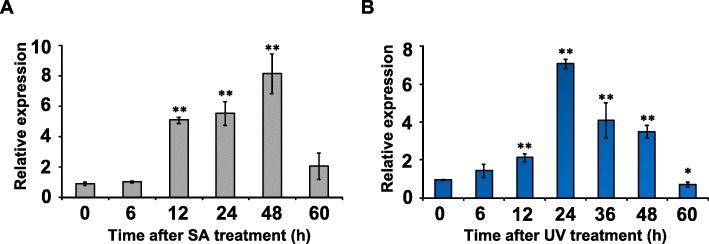
The transcriptional response of PabHLH1 to stress. Thallus was treated with SA (**a**), or exposed to UV radiation (**b**). The reference sequence used for the qRT-PCR was a gene encoding an elongation factor. The results were shown as the mean of three replicate reactions with standard deviations, *p < 0.05, ** *p* < 0.01 according to Student’s t-test

The effect of exposing *P. appendiculatum* thallus to UV irradiation or treating it with SA was to up-regulate the expression of *PabHLH1*. The SA treatment increased transcript abundance to some eight fold of the background level over the first 48 h, but the abundance of the transcript fell away sharply over the subsequent 12 h (Fig. 5a). The response to UV irradiation was less long-lived, increasing over the first 24 h, and then declining gradually (Fig. 5b).

### The sub-cellular localization and transactivation ability of PabHLH1

**Fig. 6:**
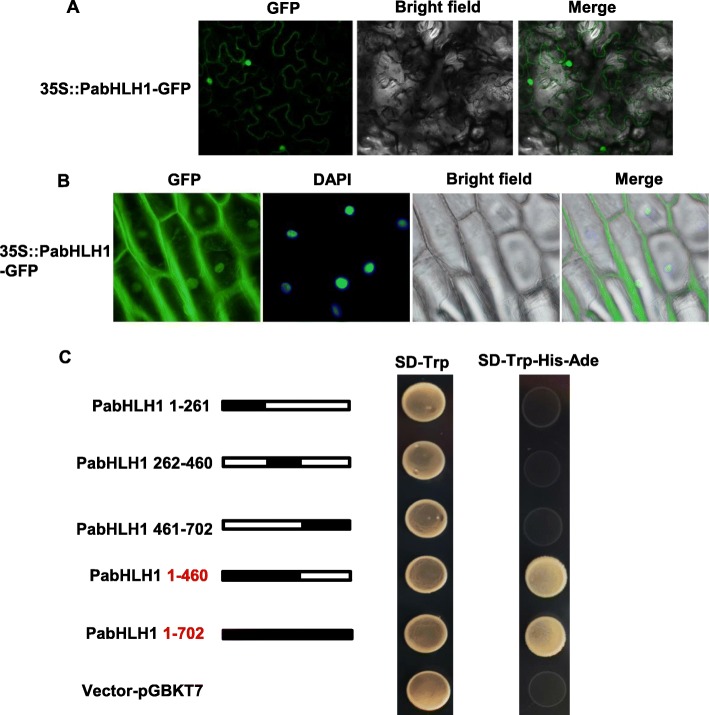
Subcellular localization and Transactivation of PabHLH1. The expression cassettes of PabHLH driven by 35S promoter, was transiently expressed in the tobacco leaves (**a**) and onion epidermal cells (**b**). The GFP fluorescence is shown in green and the DAPI fluorescence is shown in blue. **c** Four truncated fragments and the full-length protein were each introduced into pGBKT7, and transformed yeasts selected on both SD-Trp and SD-Trp-His-Ade. Construct containing PabHLH1 residues 1–460 and the full length protein was functional and supported survival on the multiple amino acid deficient medium. The black filled bar indicates the PabHLH1 domain

To analyze the subcellular localizations of PabHLH1 protein, the full-length coding sequence was fused inframe with the GFP gene and transient expressed in *N. benthamiana* leaf or onion epidermal cells. The results demonstrated that the PabHLH1 protein is deposited in the nucleus and cytoplasm (Fig. 6a, b). The transactivational activity of PabHLH1 was verified using a yeast one hybrid assay. The role of various regions of the protein was investigated by splitting the sequence into five segments (the N terminal segments formed by residues 1–261 and 1–460, and the C terminal segments 262–460, 461–702 and 1–702, see Fig. 6c). Transactivation activity was inferred when the presence of the PabHLH1 segment allowed the yeast cells to grow. On SD-Trp medium, cell growth was possible in the presence of all four segments as well as that of the empty vector, but on the SD-Trp-His-Ade medium, the cells carrying the 1–460 segments and the full-length protein 1–702 survived. The conclusion was that the transactivational activity of PabHLH1 relied on sequence lying within the segment 1–460.

### PabHLH1 over-expression increased the bibenzyls and flavonoids content of the *P. appendiculatum* thallus

**Fig. 7:**
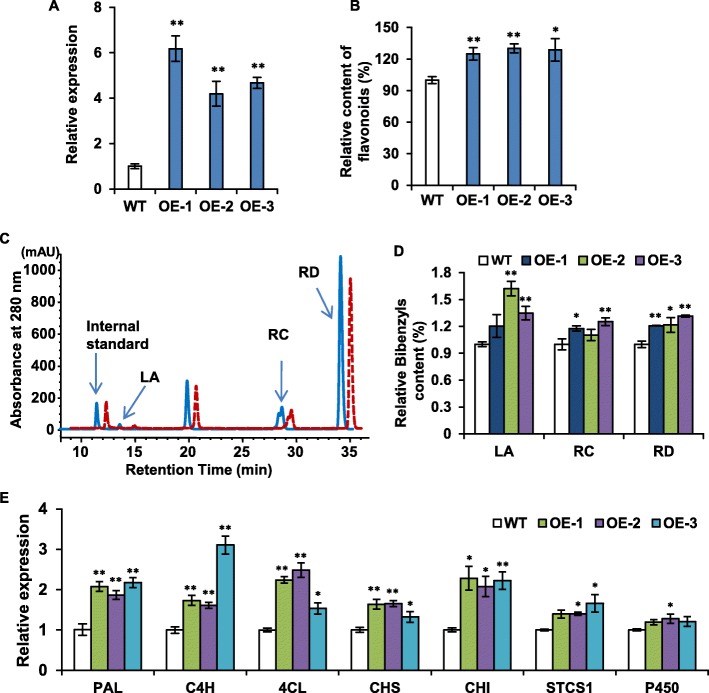
PabHLH1 transcription and biochemical analysis in transgenic *P. appendiculatum thallus*. **a** The transcription of PabHLH1 in *P. appendiculatum* wild-type (WT) and the transgenic overexpression PabHLH1 gene thallus (OE). **b** The contents of flavonoids in WT and OE thallus. **c** Representative HPLC profiles of methanolic bibenzyls extracted from OE thallus (solid line) and WT thallus (red dashed line). The major bibenzyls identified were: LA = lunularic acid, RC = riccardin C, RD = riccardin D. The internal standard was baicalein. **d** The bibenzyls contents quantified according to the HPLC analysis of WT and OE *P. appendiculatum* thallus using corresponding sdandard curve of LA, RC and RD. **e** The transcript abundance of genes encoding enzymes involved in bibenzyl and flavonoid synthesis in WT and OE thallus. Data represent mean ± S.D. with three biological repeats. *p < 0.05, ** *p* < 0.01 according to Student’s t-test

To dissect the in vivo function of PabHLH1, the gene encoding region of PabHLH1 was subcloned into the binary vector pGWB5 and transformed into *P. appendiculatum* thallus through *Agrobacterium* mediated method [26]. A qRT-PCR analysis confirmed a much higher abundance of *PabHLH1* transcript in the transgenic than in the WT thallus (Fig. 7a). Flavonoids content was determined as aglycones by preparing acid-hydrolyzed extracts. The main peak in the HPLC analysis showed the same retention time and typical UV spectrum with luteolin (Additional file 3: Figure S1). Thus, the content of flavonoids was quantified as the relative content in wild type and transgenic *P. appendiculatum* thallus and the results indicated that the transgenics accumulated more flavonoids than the wild type thallus (Fig. 7b). An HPLC separation of thallus extracts showed that the bibenzyls lunularic acid, riccardin C and riccardin D (Fig. 7c, d), identified on the basis of corresponding standard samples (Additional file 4: Figure S2), were accumulated more strongly in the transgenic than in WT thallus. The Real-time PCR results indicated that genes encoding PAL, C4H and 4CL were all significantly up-regulated in the transgenic thallus (Fig. 7e), as were those encoding the flavonoids synthesis enzymes chalcone isomerase (CHI), and chalcone synthase (CHS). The gene encoding stilbenecarboxylate synthase (STCS1) and a cytochrome P450 involved in bibenzyl synthesis, was marginally up-regulated.

### The accumulation of flavonoids and lignin in transgenic *A. thaliana*

**Fig. 8:**
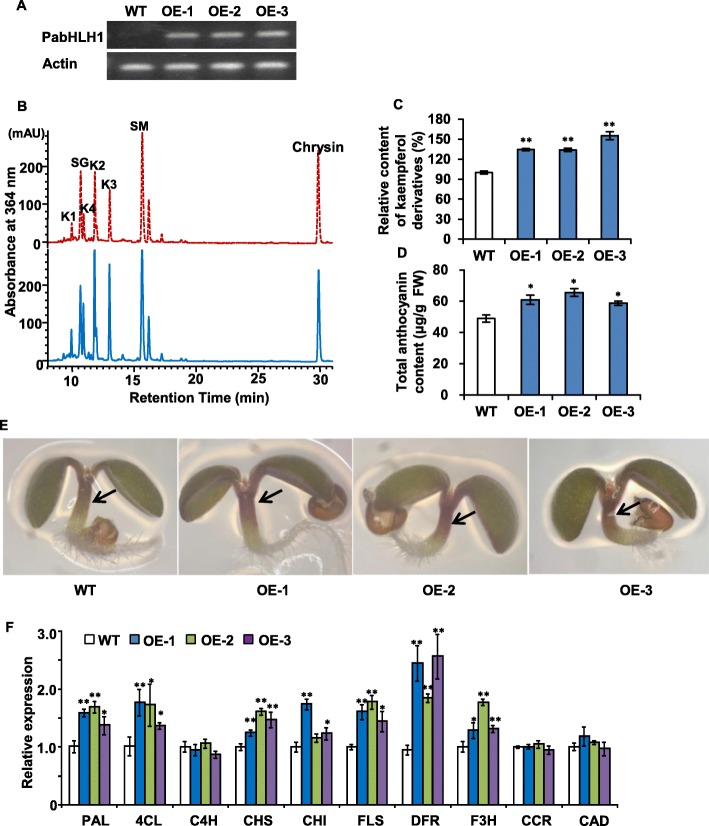
The heterologous expression of PabHLH1 in *A. thaliana * (**a**) RT-PCR analysis of expression levels of PabHLH1 in the WT and PabHLH1-OE transgenic Arabidopsis seedlings. WT, wild-type *A. thaliana*; OE: transgenic *A. thaliana* lines carrying PabHLH1. **b** Representative HPLC profiles of methanolic extracts of transgenic *Arabidopsis* seedlings line (blue solid line) and WT seedlings (red dashed line). The major flavonoid or sinapic acid derivatives are: K1, Kaempferol 3-*O*-[6″-*O*-(rhamnosyl) glucoside] 7-*O*-rhamnoside; K2, kaempferol 3-*O*-glucoside 7-*O*-rhamnoside; K3, kaempferol 3-*O*-rhamnoside 7-*O*-rhamnoside; K4, kaempferol 3-*O*-rhamnoside 7-*O*-glucoside; SM, sinapoyl malate; SG, sinapoyl glucose. The internal standard chrysin are labeled. **c** The contents of kaempferol derivatives in methanolic extracts of *Arabidopsis* seedlings by HPLC analysis. **d** The contents of anthocyanin in methanolic extracts of *Arabidopsis* seedlings. **e** Seedlings of 5 days after germination of wild type and transgenic lines OE1, OE2 and OE3*,* showing the more anthocyanin accumulation in the hypocotyl in transgenic lines indicated with arrow. **f** qRT-PCR analysis of the genes encoding key enzymes in lines OE1, OE2 and OE3. Data represent mean ± S.D. with three biological repeats. *p < 0.05, ** *p* < 0.01 according to Student’s t-test.

To ascertain the regulation functions of PabHLH1 on flavonoids biosynthesis in Arabidopsis, the construct harboring total PabHLH1 ORF in pGWB5 plasmid was transformed into *A. thaliana*. *PabHLH1* transcript was successfully identified in T_3_ plants (Fig. 8a). The analysis of three transgene homozygous plants grown on half strength MS medium for about ten days showed that the overall accumulation of flavonols derivatives was higher in the transgenics than in the WT plants (Fig. 8b, c). On the anthocyanin inducing medium, the transgenic Arabidopsis accumulated more anthocyannins than the wild type (Fig. 8d). Anthocyanin pigments accumulated in the junction of cotyledon and hypocotyl of wild type seedling less than that in the transgenic lines (Fig. 8e). This was consistent with quantification of total anthocyanin content in the seedlings of those plants (Fig. 8d). However, both the Wiesner’s staining and acetyl bromide analysis of the lignin content showed no significant differences between PabHLH1-OE and WT lines (Additional file 5: Figure S3). When the transcriptional response of the genes encoding structural enzymes responsible for the flavonoids synthesis was characterized by qRT-PCR analysis, it appeared that the genes encoding PAL, CHS, CHI, flavanone 3-hydroxylase (F3H), DFR and FLS were all up-regulated (especially so for *DFR*) (Fig. 8f). The abundance of transcript for the genes encoding cinnamoyl-CoA reductase (CCR) and cinnamyl alcohol dehydrogenase (CAD) (both these enzymes participate in lignin synthesis) was unaffected by the presence of the transgene (Fig. 8f).

## Discussion

The bHLHs represent one of the largest families of plant TFs, which regulate a wide range of plant developmental and physiological processes [12], including the biosynthesis of flavonoids. As yet, only one bHLH sequence, a positive regulator of bisbibenzyl biosynthesis, has been isolated from liverworts [26]. In the present investigation, a novel bHLH transcription factor, PabHLH1, was obtained by searching the *P. appendiculatum* transcriptomic database. Its phylogenetic relationship with bHLHs known to regulate flavonoids synthesis suggested that the gene belongs to the bHLH IIIf sub-group [41]. Analysis of its sequence revealed it has retained both the bHLH region (involved in homo- and heterodimerization) and the basic DNA-binding region [41]. In addition it has retained the His/Lys9, Glu13, and Arg17 (HER motif) residues, which confer binding to the G-box [38, 39]. Besides the bHLH domain, PabHLH1 also harbors N and C terminal domains representing the sites of interaction with myb-type TFs [19, 42]. PabHLH1 displayed both transactivational activity and was deposited in both the nucleus and cytoplasm. Basic helix-loop-helix transcript factors are expected to be localized to the nucleus. However, some bHLH proteins involved in the regulation of flavonoids biosynthesis are also cytoplasm associated [24].

The bibenzyls are a distinctive class of compounds produced by liverworts and possess a wide spectrum of biological and pharmacological activity. Like the flavonoids and lignin, they are products of the phenylpropanoid pathway, as shown by the fate of radioactively labeled precursors [9]. The synthesis of bibenzyls and flavonoids in liverworts may involve a common set of structural genes and transcriptional regulators. Here, the over-expression of PabHLH1 in *P. appendiculatum* thallus had the effect of boosting the accumulation of bibenzyls and of up-regulating genes encoding PAL, C4H, 4CL and STCS1 (Fig. 7). The content of bibenzyls and the abundance of PAL, C4H and 4CL transcript was correlated with the transcript abundance of PabHLH1. These results indicated that in *P. appendiculatum*, PabHLH1 influences bibenzyl synthesis via its up-regulation of structural genes acting early in the phenylpropanoid pathway. The increase in flavonoid content induced by PabHLH1 over-expression was accompanied by a significant up-regulation of the genes encoding the flavonoid synthesis enzymes CHI and CHS.

The constitutive expression of PabHLH1 in *A. thaliana* enhanced the accumulation of both flavonoids and anthocyanin, but had no effect on the lignin content. At the gene level, the transgenic plants experienced an up-regulation of PAL, 4CL, CHS, CHI, F3H, DFR and FLS, leaving the transcription of the lignin synthesis-associated genes CCR and CAD unaffected. DFR, the first committed enzyme of anthocyanin synthesis, was particularly strongly up-regulated (by > 2 fold). The products of both *Petunia hybrida* AN1 and AtTT8 control the expression of the flavonoid synthesis pathway structural genes CHS, CHI, F3H, FLS1 and DFR [17, 20], while the morning glory TF IpIVS regulates all these genes except for *F3H* [23]. DvIVS is a transcription factor that activates anthocyanin biosynthesis genes including DvCHS1, DvF3H, DvDFR, and DvANS in *Dahlia variabilis* [22]*.* PabHLH1 appears able to influence the expression of PAL, 4CL, CHS, CHI, F3H, DFR and FLS. These results suggested that the bHLHs transcript factors in IIIf subgroup regulate the flavonoids and anthocyanin biosynthesis through controlling the expression of several structure genes simultaneously.

A number of secondary metabolites, including the bibenzyls and flavonoids, function as abiotic stress protectants, with their synthesis commonly being triggered by environmental stresses. In some cases, the stress signal is transduced by a phytohormone, so that exogenous treatment with SA, for example, can mimic the stress response. As regulators of secondary metabolism synthesis, the bHLH TFs are inducible by stresses such as salinity and extreme temperature, as well as by treatment with phytohormone [25, 43]. Here, the transcription of PabHLH1 in *P. appendiculatum* thallus responded positively to both SA treatment and UV irradiation, as is also the case for a number of genes encoding key enzymes involved in phenylpropanoid synthesis [10, 11, 44]. The transcription of C4H and 4CL1, which encode enzymes acting in the first two steps of phenylpropanoid synthesis are known to respond positively to exogenous SA treatment [11, 45]. Meanwhile CHS, encoding the “gatekeeper” of flavonoid synthesis, is markedly induced by this treatment [44]. The indication is that PabHLH1 acts to up-regulate a series of genes which contribute to defense against abiotic stress.

## Conclusions

A bHLH TF was isolated and characterized from the liverwort *P. appendiculatum*. When over-expressed, PabHLH1 was able to regulate bisbibenzyl biosynthesis, while when constitutively expressed in *A. thaliana*, it regulated flavonoid and anthocyanin biosynthesis; in both cases, the gene acted by influencing the transcription of relevant structural genes. The functional identification of liverwort transcription factors is of significance for tracing the evolution of secondary metabolite synthesis, while potentially also suggesting strategies aimed at enhancing the production of valuable molecules in plants.

## Supplementary information

**Additional file 1: Table S1.** Sequences of oligonucleotide primers used in this study.

**Additional file 2: Table S2.** Sequences of primers used for transcription analysis.

**Additional file 3: Figure S1.** The flavonoids analysis of the WT and PabHLH1-OE transgenic *P. appendiculatum* thallus. (A) WT, (B) OE-1 transgenic line, (C) OE-2 transgenic line, and (D) luteolin standards. (E) UV spectra of P1 and (F) UV spectra of luteolin. The internal standard myricetin is labeled.

**Additional file 4: Figure S2.** HPLC profiles of bibenzyls from PabHLH1 transgenic *P. appendiculatum* thallus (D) and the corresponding standards (A-C). (A) The internal standard is baicalein; (B) LA, Lunularic acid and RC, Riccardin C; (C) RD, Riccardin D. UV spectra of (E) LA, (G) RC, and (I) RD standard; UV spectra of the bibenzyls extracted from PabHLH1 transgenic line, LA (F), RC (H) and RD (J).

**Additional file 5: Figure S3.** Lignin analysis of the *A. thaliana* with heterologous expression of PabHLH1. (A) One vascular bundle in the frozen sections of the stems stained with Wiesner’s method. (B) Quantification of lignin content. WT, wild-type *A. thaliana*; OE: transgenic *A. thaliana* lines carrying PabHLH1.

## Data Availability

The data supporting the results presented in this article are included as additional files. Further requests for materials should be addressed to A.-X.C (aixiacheng@sdu.edu.cn).

## Abbreviations

4CL: 4coumarate: coenzyme A ligase
ANS: Anthocyanidin synthase
C4H: Cinnamic acid 4-hydroxylation
CHI: Chalcone isomerase
CHS: Chalcone synthase
DBR: Double bond reductase
DFR: Dihydroflavonol reductase
F3H: Flavanone 3β-hydroxylase
FLS: Flavonol synthase
PAL: Phenylalanine ammonia lyase
STCS: Stilbenecarboxylate synthase
