# Supplementary material for: Functional characterization of a liverworts bHLH transcription factor involved in the regulation of bisbibenzyls and flavonoids biosynthesis
Source: BMC Plant Biol. 2019 Nov 14;19:497. doi: 10.1186/s12870-019-2109-z (PMC6854758; doi:10.1186/s12870-019-2109-z)
Supplement: Supplementary file 1 — Additional file 1: Table S1. Sequences of oligonucleotide primers used in this study. [file 12870_2019_2109_MOESM1_ESM.docx]

**Suppl Table 1** Sequences of oligonucleotide primers used in this study.

| Primer name | Primer sequences 5′-3′ |
| --- | --- |
| PabHLH1-GSP | GAAGGCGACAATGAGGGGCCAACT |
| PabHLH1-NGSP | TGGGGATGCAGAGGACGAGACAAC |
| PabHLH1-F | CGTTTGCGAGTGAATGTGCT |
| PabHLH1-R | GCTAACCAGTATGCTCTTTC |
| PabHLH1-attB1-F | GGGGACAAGTTTGTACAAAAAAGCAGGCTTAACCATGGCTGGAGCGGGGAAAAG |
| PabHLH1-attB2-R | GGGGACCACTTTGTACAAGAAAGCTGGGTCTCTGGTTTCCGTCCCAGCAG |
| PabHLH1-attB2-OER | GGGGACCACTTTGTACAAGAAAGCTGGGTCTTATCTGGTTTCCGTCCCAG |
| PabHLH1-vector-F | AGGAAAGGCCATCGTTGAAG |
| PabHLH1-gene-R | TAGCCTCATTGGTCTCTGTC |
| PabHLH1(1-261)-NdeI-1F | GGAATTCCATATGATGGCTGGAGCGGGGAAAAG |
| PabHLH1(1-261)-BamHI-1R | CGGGATCCCGAATCTTGGTGGTCAACAT |
| PabHLH1(262-460)-NcoI-2F | CATGCCATGGCAATGGTCACTTTCTCGAACGA |
| PabHLH1(262-460)-BamHI-2R | CGGGATCCATTCAGCTTCTCCCTACGAC |
| PabHLH1(461-702)-NdeI-3F | GGAATTCCATATGGATAGGTTCATGTCCCTCCG |
| PabHLH1(461-702)-EcoRI-3R | CGGAATTCTCTGGTTTCCGTCCCAGCAG |
| PabHLH1(1-460)-NcoI-4F | CATGCCATGGCAATGGCTGGAGCGGGGAAAAG |
| PabHLH1(1-460)-BamHI-4R | CGGGATCCATTCAGCTTCTCCCTACGAC |
| PabHLH1-NcoI-F | CATGCCATGGCGATGGCTGGAGCGGGGAAAAG |
| PabHLH1-XhoI-R | CCCTCGAGTTATCTGGTTTCCGTCCCAG |
| PabHLH1-a957c-F | CATCTCGCTCGATCCCTATGTAGTAGGTGG |
| PabHLH1-a957c-R | CCACCTACTACATAGGGATCGAGCGAGATG |
| PabHLH1-NdeI-5F | GGAATTCCATATGATGGCTGGAGCGGGGAAAAG |
| PabHLH1-NcoI-5R | CATGCCATGGTTATCTGGTTTCCGTCCCAG |
