# Supplementary material for: Functional characterization of a liverworts bHLH transcription factor involved in the regulation of bisbibenzyls and flavonoids biosynthesis
Source: BMC Plant Biol. 2019 Nov 14;19:497. doi: 10.1186/s12870-019-2109-z (PMC6854758; doi:10.1186/s12870-019-2109-z)
Supplement: Supplementary file 2 — Additional file 2: Table S2. Sequences of primers used for transcription analysis. [file 12870_2019_2109_MOESM2_ESM.docx]

**Suppl Table 2** Sequences of primers used for transcription analysis.

| Primer name | | Primer sequences 5′-3′ |
| --- | --- | --- |
| PabHLH1-qRT-F | | TTTAGCGCAAGGACGACATG |
| PabHLH1-qRT-R | | CAATAGCAGGATCTTCACGC |
| *Plagiochasma appendiculatum* | elongation factor | TATTGCTCTGTGGAAGTTTG  TCATCTGCTTCACTCCCAAC |
|  | PAL | GATTTCTTAATGCAGGCGTG  GGAGTAATGTGCTTGTTCAG |
|  | 4CL | TGGACAGGGGTATGGTATGA  CTTGCCGTGAGGAAGTGAGA |
|  | C4H | GAGCACTGGAGAAGAATGAG  GCTGCAACCTTCTTCTGATG |
|  | CHS | ATTGATGGTCTCGTCGGATC  TGAGCAAATGGAAGACGAGG |
|  | CHI | TGATCTGGGAGTCGTTGATC  AGTCTGCCTCCGTATTATCC |
|  | DFR | TCCTACTTCGCTATCGGTCC  GGTTTCCTGCTGTTCGTTTG |
|  | STCS | AGAGAAGGCAGCAATCGAGG  CCCTTGTTGTTCTCAGCCAG |
|  | P450 | ATGTGTCTGATGATGTTCGG  GATTCTCACAACGGGCTCTG |
| *Arabidopsis thaliana* | Actin | GTCTGGATCGGAGGATCAAT  CCTGTGAACAATCGATGGAC |
|  | PAL | CGCTCTTCGTACTTCTCCTC  GCTATCGCCAATCTCGTGTT |
|  | C4H | TGATTCTCGCCACGGTGATT  TGAGGAGACGACGACTAGGT |
|  | 4CL | CTTGTACCGCCGCTAGTGAT  TCCTGCCTCCGTCATACCAT |
|  | CHS | GTGATGGCTGGTGCTTCTTC  ACTGTTGGTGATGCGGAAGT |
|  | CHI | CGCCGTTCCTTCTCTATCTG  CCACACAATTCTCCGTCACT |
|  | FLS | ACTATTATCCGCCGTGTCCT  TCGCCGATGTGAACAATGAC |
|  | DFR | CTTCGGGTTTCATCGGTTCAT  CATCGTAGCTTCCTTCCTCAG |
|  | F3H | GTTGAGGCTTGTGAGAATTGG  CTGGAGGTGACTAGAGACGAT |
|  | CCR | GCCACACCGATCTTCATCAA  CTACAACCTCCGCAACATCC |
|  | CAD | GCCACACCGATCTTCATCAA  CTACAACCTCCGCAACATCC |
