# Supplementary material for: Functional characterization of a liverworts bHLH transcription factor involved in the regulation of bisbibenzyls and flavonoids biosynthesis
Source: BMC Plant Biol. 2019 Nov 14;19:497. doi: 10.1186/s12870-019-2109-z (PMC6854758; doi:10.1186/s12870-019-2109-z)
Supplement: Supplementary file 3 — Additional file 3: Figure S1. The flavonoids analysis of the WT and PabHLH1-OE transgenic P. appendiculatum thallus. (A) WT, (B) OE-1 transgenic line, (C) OE-2 transgenic line, and (D) luteolin standards. (E) UV spectra of P1 and (F) UV spectra of luteolin. The internal standard myricetin is labeled. [file 12870_2019_2109_MOESM3_ESM.pdf]

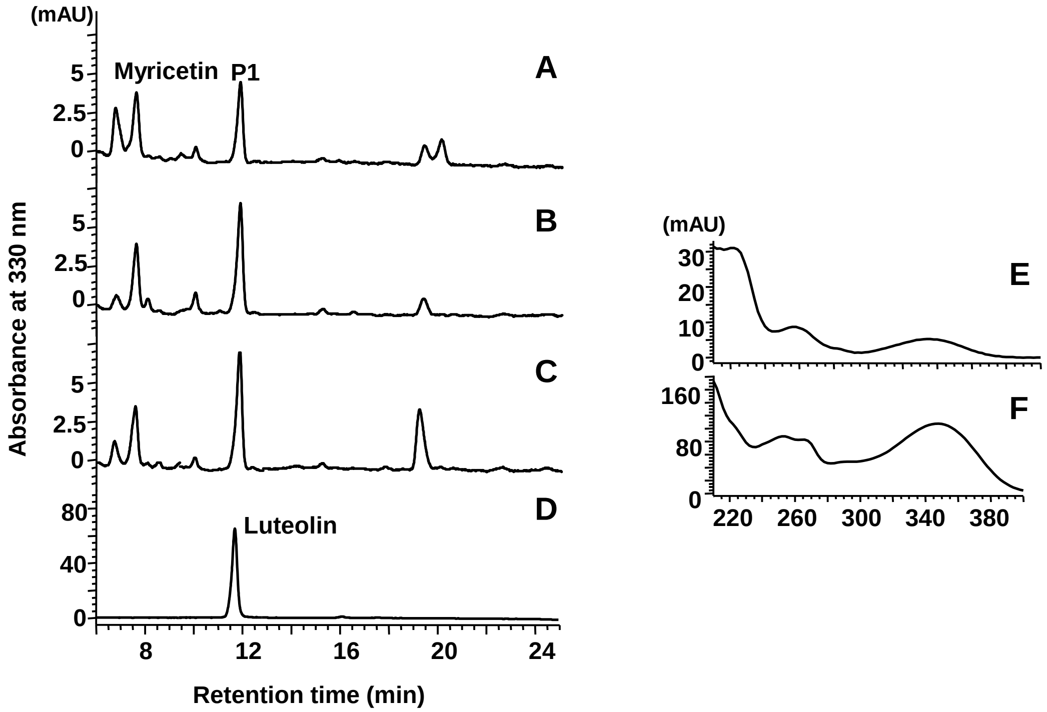

Figure S1 The flavonoids analysis of the WT and PabHLH1-OE transgenic *P. appendiculatum* thallus. (A) WT, (B) OE-1 transgenic line, (C) OE-2 transgenic line, and (D) luteolin standards. (E) UV spectra of P1 and (F) UV spectra of luteolin. The internal standard myricetin is labeled.
