# Supplementary material for: Functional characterization of a liverworts bHLH transcription factor involved in the regulation of bisbibenzyls and flavonoids biosynthesis
Source: BMC Plant Biol. 2019 Nov 14;19:497. doi: 10.1186/s12870-019-2109-z (PMC6854758; doi:10.1186/s12870-019-2109-z)
Supplement: Supplementary file 4 — Additional file 4: Figure S2. HPLC profiles of bibenzyls from PabHLH1 transgenic P. appendiculatum thallus (D) and the corresponding standards (A-C). (A) The internal standard is baicalein; (B) LA, Lunularic acid and RC, Riccardin C; (C) RD, Riccardin D. UV spectra of (E) LA, (G) RC, and (I) RD standard; UV spectra of the bibenzyls extracted from PabHLH1 transgenic line, LA (F), RC (H) and RD (J). [file 12870_2019_2109_MOESM4_ESM.pdf]

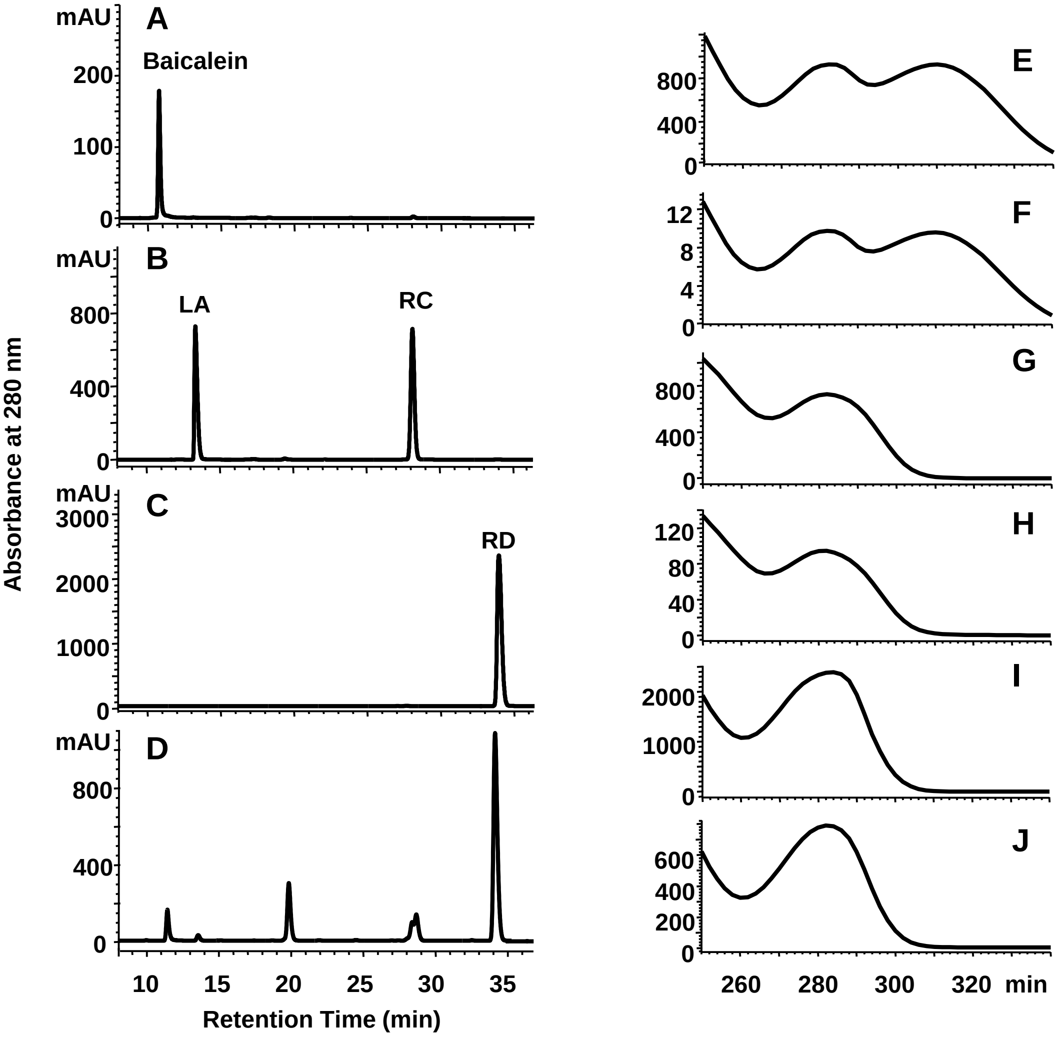

Figure S2 HPLC profiles of bibenzyls from *PabHLH1* transgenic *P. appendiculatum* thallus (D) and the corresponding standards (A-C). (A) the internal standard is baicalein, (B) LA, Lucularic acid and RC, Riccardin C; (C) RD, Riccardin D. UV spectra of (E) LA, (G) RC, and (I) RD standard; UV spectra of the bibenzyls extracted from *PabHLH1* transgenic line, LA (F), RC (H) and RD (J).
