# Supplementary material for: Functional characterization of a liverworts bHLH transcription factor involved in the regulation of bisbibenzyls and flavonoids biosynthesis
Source: BMC Plant Biol. 2019 Nov 14;19:497. doi: 10.1186/s12870-019-2109-z (PMC6854758; doi:10.1186/s12870-019-2109-z)
Supplement: Supplementary file 5 — Additional file 5: Figure S3. Lignin analysis of the A. thaliana with heterologous expression of PabHLH1. (A) One vascular bundle in the frozen sections of the stems stained with Wiesner’s method. (B) Quantification of lignin content. WT, wild-type A. thaliana; OE: transgenic A. thaliana lines carrying PabHLH1. [file 12870_2019_2109_MOESM5_ESM.pdf]

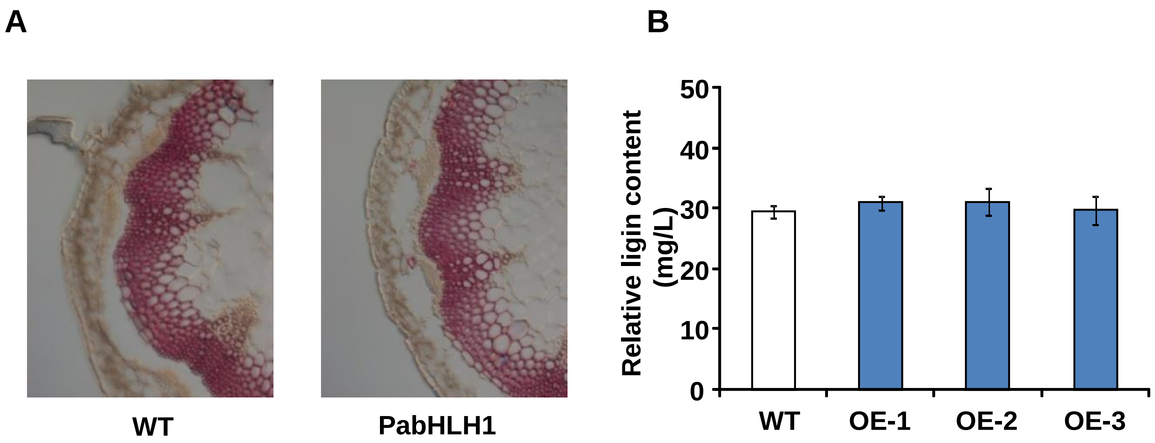

Figure S3 Lignin analysis of the *A. thaliana* with heterologous expression of *PabHLH1.*

(A) One vascular bundle in the frozen sections of the stems stained with Wiesner's method.

(B) Quantification of lignin content. Lignin content was calculated by cell-wall residue. WT, wild-type *A. thaliana* ecotype Columbia; OE: transgenic *A. thaliana* lines carrying PabHLH1. Data are the mean value ± standard deviation of more than three biological repeats.
